# Crystal structure and Hirshfeld surface analysis of the cocrystal formed between 2,3-di­amino­pyrazine and 2,3,5,6-tetra­fluoro­terephthalic acid

**DOI:** 10.1107/S2056989026001581

**Published:** 2026-02-24

**Authors:** Eric Bosch

**Affiliations:** a Department of Chemistry and Biochemistry Missouri State University, 901 South National Avenue Springfield MO 65897 USA; Texas A & M University, USA

**Keywords:** crystal structure, hydrogen bond, supra­molecular network, pyrazinium, tetra­fluorophthalic acid

## Abstract

The cocrystal formed between 2,3-di­amino­pyrazine and 2,3,5,6-tetra­fluoro­phthalic acid, crystallizes as the solvated salt bis­(2,3-di­amino­pyrazin-1-ium) 2,3,5,6-tetra­fluoro­phthalate–2,3,5,6-tetra­fluoro­phthalic acid (1/1), 2C_4_H_7_N_4_^+^·C_8_F_4_O_4_^2−^·C_8_H_2_F_4_O_4_, in the triclinic space group *P*1 with one unique protonated 2,3-di­amino­pyrazinium cation, one half a tetra­fluoro­phthalic acid mol­ecule and one half of a tetra­fluoro­phthalate anion. The cocrystal forms a supra­molecular network with cooperative neutral and charge-assisted hydrogen bonding. In this, the linear network of alternating pyrazinium and tetra­fluoro­phenyl moieties is crosslinked through bifurcated hydrogen bonds of two amino H atoms and a carboxyl oxygen to form a corrugated two-dimensional network.

## Abstract 2

The cocrystal formed between 2,3-di­amino­pyrazine and tetra­fluoro­phthalic acid, C_24_H_16_F_8_N_8_O_8_, crystalizes as a salt in the triclinic space group *P*1 with one unique protonated 2,3-di­amino­pyrazinium cation, one half a tetra­fluoro­phthalic acid mol­ecule and one half of a tetra­fluoro­phthalate anion. The salt forms a corrugated two-dimensional supra­molecular network with cooperative neutral and charge assisted hydrogen bonding.

## 1. Chemical context

Hydrogen-bonded networks are well-established and rely on the choice of hydrogen-bond (HB) donors and HB acceptors capable of forming multiple HB’s to facilitate formation of a desired network. Recent examples of this include the use of hydrogen-bonded supra­molecular networks for the selective removal of perchlorate anion from aqueous media (Tian *et al.*, 2025 ▸) and the application of hydrogen-bonding inter­actions to facilitate selective hydrogenation (Shi *et al.*, 2023 ▸). Wang and coworkers investigated the formation of hydrogen-bonded supra­molecular assemblies on cocrystallization with a series of aza compounds (Wang *et al.*, 2013 ▸). Earlier we also reported on the cooperative charge-assisted N—H⋯O and C—H⋯N hydrogen bonding on cocrystals formed between tetra­fluoro­benzoic acid and 2-amino pyrazine (Bosch & Bowling, 2020 ▸). Here we report the cocrystallization of tetra­fluoro­phthalic acid (TPA), formally a ditopic hydrogen-bond donor, with 2,3-di­amino­pyrazine (DAP), potentially a ditopic hydrogen-bond acceptor and hydrogen-bond donor, with the expectation that a linear supra­molecular polymer would be formed. The 1:1 cocrystal comprises a more complex hydrogen-bonded network through neutral and charge-assisted hydrogen bonding.
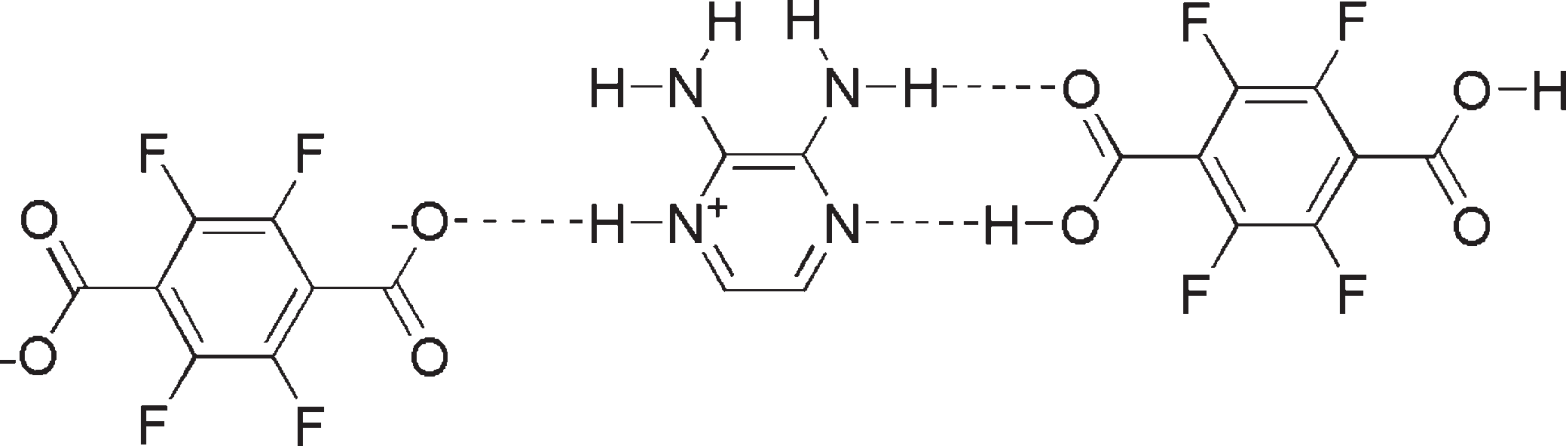


## 2. Structural commentary

**Figure 1:**
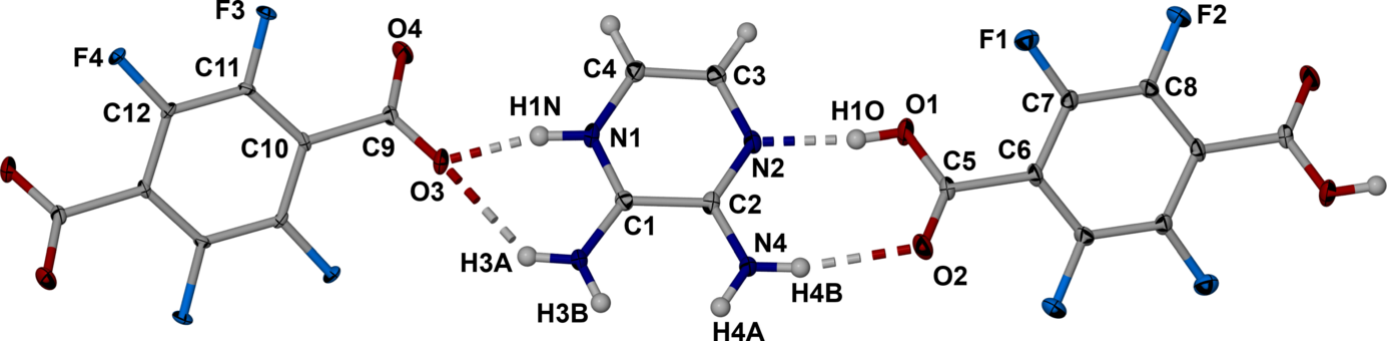
Labelled asymmetric unit of the cocrystal formed between DAP and TPA with hydrogen bonds shown as dashed lines. Displacement ellipsoids drawn at the 50% level.

The cocrystal formed between 2,3,5,6-tetra­fluoro­terephthalic acid and 2,3-diamino­pyrazine crystallized in the triclinic space group *P*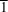 with one mol­ecule of DAP and half of a TPA mol­ecule along with one half of a deprotonated TPA mol­ecule in the asymmetric unit as shown in Fig. 1 ▸. It is noteworthy that the C—O bond distances C9—O3 and C9—O4 are 1.257 (5) and 1.248 (5) Å respectively which is consistent with a phthalate moiety. Additionally, C—O bond distances for C5—O1 and C5—O2 are 1.305 (5) and 1.220 (5) Å respectively, consistent with the C—O single bond and double bond of phthalic acid. It is noteworthy that the carboxyl­ate and the carb­oxy­lic acid moieties are both significantly twisted out of the plane of the tetra­fluoro­benzene rings at angles of 44.3 (4) and 45.0 (4)°, respectively. This rotation allows the carb­oxy­lic acid and carboxyl­ate moieties to be almost coplanar with the central pyrazinium cation with torsional angles of only 6.5 (3) and 7.3 (2)°, respectively.

The carboxyl­ate moiety forms a charge-assisted bifurcated hydrogen bond to the pyrazinium hydrogen and the adjacent amine hydrogen H3*A* with O3⋯H1 and O3⋯H3*A* distances of 1.73 (3) and 2.30 (7) Å, respectively. This inter­action has graph set notation 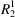
(6). In contrast the carboxyl moiety, as hydrogen bond donor and acceptor, forms two cooperative hydrogen bonds to the amino­pyrazine moiety as complementary hydrogen bond acceptor and donor with graph-set notation of 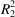
(8). The N2⋯H1*A* and O2⋯H4*A* separations are 1.60 (8) and 2.02 (6) Å, respectively. Complete details of these HB’s and others in the structure are collated in Table 1 ▸.

## 3. Supra­molecular features

**Figure 2:**
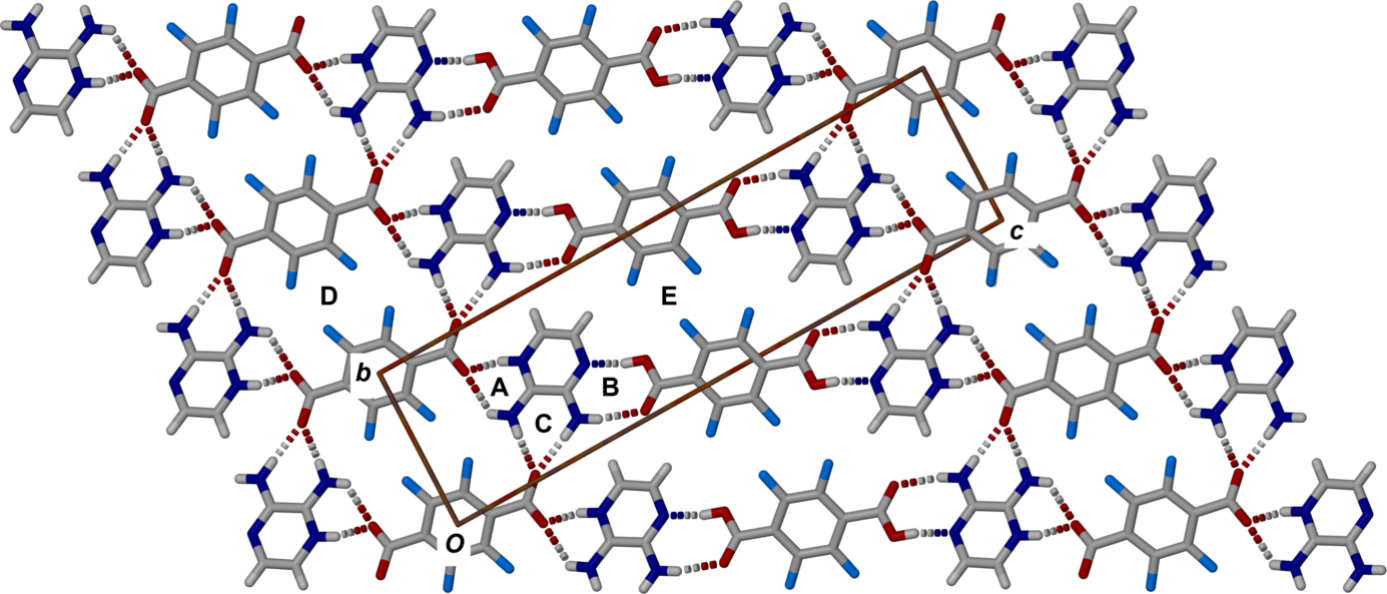
View along the *a* axis of the cocrystal showing four adjacent strands within the extended hydrogen-bonded network to illustrate the crosslinking between adjacent strands. The two cyclic hydrogen-bonded motifs from Fig. 1 ▸ are labelled A and B. The crosslinking bifurcated hydrogen-bond motif is labelled C. The larger hydrogen-bonded rings are labelled D and E (see text).

**Figure 3:**
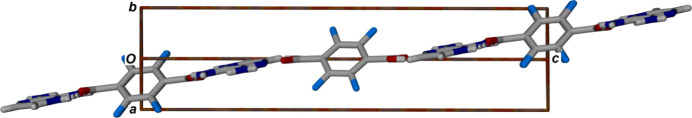
View along (110) of a portion of the packing within the unit cell of the cocrystal showing a side view of three layers of the planar hydrogen-bonded network in Fig. 2 ▸. illustrating the twist between the tetra­fluoro­benzene and pyrazinium moieties.

The two hydrogen-bond networks in Fig. 1 ▸, labelled A and B in Fig. 2 ▸, result in infinite linear chains of alternating tetra­fluoro­phthalate–pyrazinium–tetra­fluoro­phthalic acid–pyrazin­ium moieties. These chains are crosslinked through a bifurcated hydrogen bond from the second carboxyl­ate O atom, O4, to a hydrogen from each of the amino groups, H3*B* and H4*B*, with graph set notation 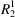
(7). This is shown as C in Fig. 2 ▸ which shows the resultant two-dimensional network. The oxygen–hydrogen separations are 1.98 (6) and 1.88 (6) Å for O4⋯H3*B* and O4⋯H4*A*, respectively. This crosslinking sets up two larger hydrogen-bonded rings, D and E respectively, shown in Fig. 2 ▸ with graph set notation 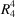
(22) and 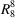
(40). There is a close C—H⋯O contact between H4 and O2 with an H⋯O separation of 2.61Å. The two-dimensional hydrogen-bonded network is a corrugated plane with the tetra­fluoro­phenyl rings tilted with respect to the planes of the pyrazinium moieties (Fig. 3 ▸). Complete details of the HB’s in the cocrystal structure are collated in Table 1 ▸.

These planar hydrogen-bonded supra­molecular networks are π-stacked as shown viewed along the line (110) in Fig. 3 ▸ with offset π-stacks of each of the three components. The tetra­fluoro­phthalic acid and tetra­fluoro­phthalate moieties are twisted out of the plane of the pyrazinium mol­ecules to accommodate the fluorine atoms. The perpendicular distances between the offset stacks of pyrazinium, tetra­fluoro­phthalic acid and tetra­fluoro­phthalate mol­ecules are 3.243 (2), 3.430 (2) and 3.429 (2) Å with slippages of 1.671, 1.242 and 1.245 Å, respectively.

**Figure 4:**
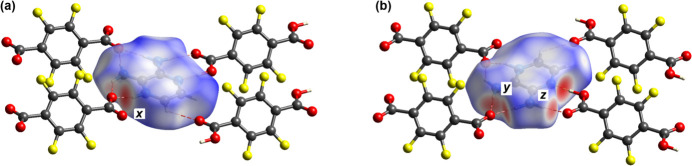
Two views of the Hirshfeld surface for the pyrazinium cation within the cocrystal with *d*_norm_ mapped over the surface. Red areas indicate contacts significantly closer than the sum of the respective van der Waals radii. Dashed lines showing atom-to-atom close contacts. (*a*) the ‘*x’* label shows the carboxyl­ate pyrazinium H⋯O contact; (*b*) the ‘*y’* corresponds to the carboxyl­ate hydrogen bond to the two amino groups and ‘*z’* corresponds to the two-pronged hydrogen bond of the carb­oxy­lic acid to the amino­pyrazine.

The program *CrystalExplorer21* (Spackman *et al.*, 2021 ▸) was used to calculate and plot the Hirshfeld surface of each mol­ecule within the cocrystal. The surface colouration is a visual representation of the inter­molecular atom-to-atom separation as compared to the sum of the van der Waals radii with close contacts coloured red. Fig. 4 ▸ shows two views of the Hirshfeld surface of the pyrazinium mol­ecule within the cocrystal. The adjacent mol­ecules responsible for close contacts are correlated with hydrogen bonding inter­actions shown. The charge-assisted hydrogen-bond inter­action is labelled ‘*x*’ in Fig. 4 ▸(*a*), the bifurcated inter­action between O and the two NH_2_ groups labelled ‘*y*’ and the two-pronged carb­oxy­lic acid hydrogen-bonding inter­action is labelled ‘*z*’ [Fig. 4 ▸(*b*)].

**Figure 5:**
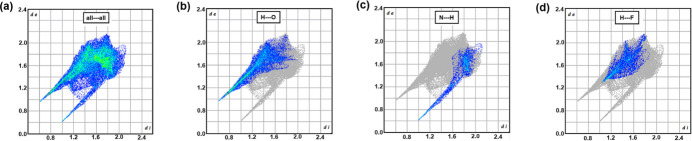
Two-dimensional fingerprint plots showing the contributions of the major inter­actions to the total Hirshfeld surface area of the pyrazinium moiety⋯F. The first atom listed in (*b*) to (*d*) corresponds to a pyrazinium atom.

Fingerprint analysis of the Hirshfeld surface of the pyrazinium cation allows the inter­actions to be separated according to the atom within the surface and the inter­acting atom outside the surface as shown in Fig. 5 ▸. In these plots the most common inter­actions are bright green and the least common inter­actions are dark blue. Thus in Fig. 5(*a*), which shows all atom-to-atom inter­actions, the most common inter­actions are centred in the area defined by atom-to-atom separation between 3.2 to 4.0 Å typical of π-stacked aromatics. The breakdown to H⋯O, H⋯H and H⋯F contacts, shown in Fig. 5(*b*), (*c*) and (*d*) ▸, highlights the closest contacts as H⋯O and N⋯H with the narrow spikes.

Breakdown of these contacts element-to-element to the pyrazinium cation revealed that the H⋯O inter­action dominated, corresponding to 27.8% of the surface area, while the N⋯H inter­action corresponds to 11.0% of the surface area of the pyrazinium cation [Fig. 5(*b*) and (*c*) ▸]. The breakdown of atom-to-atom inter­actions to the surface of the two tetra­fluoro moieties, collated in Table 2 ▸, highlights the dominance of O⋯H hydrogen bonding especially the carboxyl­ate oxygen atoms. The H⋯H inter­actions correspond to the π-stacked pyrazinium inter­actions.

Similar fingerprint analysis of the Hirshfeld surface of each of the tetra­fluoro­phthalate and tetra­fluoro­phthalic acid moieties within the cocrystal was performed and the results are collated in Table 3 ▸. As expected, the O⋯H contribution is larger for the phthalate than the phthalic acid and dominates. F⋯F and C⋯C inter­actions correspond to π-stacked mol­ecules, while the F⋯H inter­actions are inter­planar mol­ecular inter­actions.

## 4. Database survey

A search of the Cambridge Structural Database (CSD, Version 6.0.1, Nov 2025; Groom *et al.*, 2016 ▸) using Conquest (Version 2025.3.0, Build 466532; Bruno *et al.*, 2002 ▸) for structures containing the 2,3-di­amino­pyrazine did not yield any structures. Indeed, there were only four structures that include the 2,3-di­amino­pyrazine core. These include a structure of the di­cyano derivative 5,6-di­aminopyrazine-2,3-dicarbo­nitrile (refcode NUZMON; Semenov *et al.*, 2020 ▸) as well as three structures with substituted 2,3-diaminoquinoxaline core. Inter­estingly one of these structures (refcode DURSER; Yuan *et al.*, 2020 ▸) is a 1:1 cocrystal salt formed between 2,3-diaminquinoxaline and benzene-1,3,5-tri­carb­oxy­lic acid in which the quinoxaline is protonated and one carb­oxy­lic acid moiety deprotonated. A search of the database for organic only structures that include tetra­fluoro­phthallate and tetra­fluoro­phthallic acid yielded seven unique structures [refcodes AGIJEJ (Mali *et al.*, 2023 ▸), HIQVUC (Xiao *et al.*, 2023 ▸), REXMUE, REXNIT, REXNOZ (Wang *et al.*, 2013 ▸), YOCZOH and YOCZUN (Wang *et al.*, 2014 ▸)]. The carboxyl­ate C—O bond distances range from 1.212 to 1.273 Å with an average of 1.246 Å while the carb­oxy­lic acid C—O distances range from 1.285 to 1.306 Å with an average of 1.296 Å and C=O distances range from 1.196 to 1.214 Å with an average of 1.206 Å. The C—O distances reported herein are consistent with this data.

## 5. Synthesis and crystallization

2,3-Di­amino­pyrazine and 2,3,5,6-tetra­fluoro­terephthalic acid were used as supplied. An equimolar amount (0.1 mmol) of each component was added to a screw-capped vial and 4 mL of ethanol added and the solution was gently heated, resulting in the formation of a homogeneous mass of crystals after 2 weeks.

## 6. Refinement

Crystal data, data collection and structure refinement details are summarized in Table 4 ▸. All hydrogen atoms were observed in the difference maps during refinement and added to C as riding atoms in geometrically idealized positions. The pyrazinium proton was restrained in the refinement with N—H = 0.87 (2) Å and with *U*_iso_(H) = 1.2*U*_eq_(N). The carb­oxy­lic acid proton was restrained in the refinement with O—H = 0.84 (2) Å and with *U*_iso_(H) = 1.2*U*_eq_(O). In the difference map of the final solution, the residual peaks correspond to a minor disorder component. Attempts to resolve this disorder with a free variable were unsuccessful.

## Supplementary Material

Crystal structure: contains datablock(s) I. DOI: 10.1107/S2056989026001581/jy2069sup1.cif

Structure factors: contains datablock(s) I. DOI: 10.1107/S2056989026001581/jy2069Isup2.hkl

Supporting information file. DOI: 10.1107/S2056989026001581/jy2069Isup3.cdx

CCDC reference: 2531359

Additional supporting information:  crystallographic information; 3D view; checkCIF report

## Figures and Tables

**Table 1:** Hydrogen-bond geometry (Å, °)

*D*—H⋯*A*	*D*—H	H⋯*A*	*D*⋯*A*	*D*—H⋯*A*
C3—H3⋯F2^i^	0.95	2.59	3.416 (5)	145
C4—H4⋯O2^ii^	0.95	2.61	3.307 (5)	130
N4—H4*A*⋯O2	0.96 (6)	2.02 (6)	2.980 (4)	175 (5)
N1—H1⋯O3	0.88 (2)	1.73 (3)	2.595 (4)	165 (7)
N4—H4*B*⋯O4^iii^	0.94 (6)	1.88 (6)	2.814 (4)	177 (5)
N3—H3*A*⋯F3^iv^	1.01 (7)	2.54 (7)	3.027 (4)	109 (5)
N3—H3*A*⋯O3	1.01 (7)	2.30 (7)	3.091 (4)	134 (5)
N3—H3*A*⋯F4^v^	1.01 (7)	2.39 (7)	3.249 (4)	143 (6)
O1—H1*A*⋯N2	1.02 (8)	1.60 (8)	2.589 (4)	162 (7)
N3—H3*B*⋯O4^iii^	0.96 (6)	1.89 (6)	2.855 (5)	174 (5)

Symmetry codes: (i) 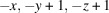; (ii) 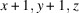; (iii) 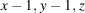; (iv) 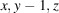; (v) 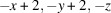.

**Table 2:** Fingerprint analysis for the pyrazinium cations within the cocrystal

Inter­action	H⋯O^*a*^	N⋯H	H⋯F	H⋯H	H⋯C	N⋯C
Percentage	27.8	11.0	13.7	22.0	9.8	8.1

(*a*) First element corresponds to an element within the pyrazinium cation, and the second element is the close atom outside of the pyrazinium cation Hirshfeld surface.

**Table 3:** Fingerprint analysis for each of the tetra­fluoro­phthalate and tetra­fluoro­phthalic acid moieties within the cocrystal.

Inter­action/Moiety	O⋯H^*a*^	F⋯F	F⋯H	C⋯C	H⋯N
Tetra­fluoro­phalate	33.2	19.9	14.6	10.8	–
Tetra­fluoro­phthalic acid	18.2	19.0	10.2	10.0	6.5

(*a*) First element corresponds to an element within the tetra­fluoro moiety Hirshfeld surface, and the second element is the close atom outside of the tetra­fluoro­phenyl moiety Hirshfeld surface.

**Table 4:** Experimental details

Crystal data	
Chemical formula	2C_4_H_7_N_4_^+^·C_8_F_4_O_4_^2−^·C_8_H_2_F_4_O_4_
*M*_r_	696.45
Crystal system, space group	Triclinic, *P* 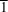
Temperature (K)	100
*a*, *b*, *c* (Å)	3.6482 (9), 6.851 (2), 24.959 (6)
α, β, γ (°)	88.125 (5), 88.452 (3), 86.426 (3)
*V* (Å^3^)	622.0 (3)
*Z*	1
Radiation type	Mo *K*α
μ (mm^−1^)	0.18
Crystal size (mm)	0.47 × 0.40 × 0.10

Data collection	
Diffractometer	Bruker APEXI CCD
Absorption correction	Multi-scan (*SADABS*; Krause *et al.*, 2015 ▸)
*T*_min_, *T*_max_	0.649, 0.746
No. of measured, independent and observed [*I* > 2σ(*I*)] reflections	7403, 2824, 2661
*R*_int_	0.022
(sin θ/λ)_max_ (Å^−1^)	0.647

Refinement	
*R*[*F*^2^ > 2σ(*F*^2^)], *wR*(*F*^2^), *S*	0.086, 0.212, 1.21
No. of reflections	2824
No. of parameters	241
No. of restraints	1
H-atom treatment	H atoms treated by a mixture of independent and constrained refinement
Δρ_max_, Δρ_min_ (e Å^−3^)	0.96, −0.53

Computer programs: *APEX2* and *SAINT* (Bruker, 2014 ▸), *SHELXT2018/2* (Sheldrick, 2015*a* ▸), *SHELXL2019/2* (Sheldrick, 2015*b* ▸) and *X-SEED4* (Barbour, 2020 ▸).

## supplementary crystallographic information

### (I) Crystal data

**Table:**

2C_4_H_7_N_4_^+^·C_8_F_4_O_4_^2^^−^·C_8_H_2_F_4_O_4_	*Z* = 1
*M**_r_* = 696.45	*F*(000) = 352
Triclinic, *P*1	*D*_x_ = 1.859 Mg m^−^^3^
*a* = 3.6482 (9) Å	Mo *K*α radiation, λ = 0.71073 Å
*b* = 6.851 (2) Å	Cell parameters from 3862 reflections
*c* = 24.959 (6) Å	θ = 2.5–27.4°
α = 88.125 (5)°	µ = 0.18 mm^−^^1^
β = 88.452 (3)°	*T* = 100 K
γ = 86.426 (3)°	Irregular block, clear colourless
*V* = 622.0 (3) Å^3^	0.47 × 0.40 × 0.10 mm

### (I) Data collection

**Table:**

Bruker APEXI CCD diffractometer	2661 reflections with *I* > 2σ(*I*)
Detector resolution: 8.3660 pixels mm^-1^	*R*_int_ = 0.022
φ and ω scans	θ_max_ = 27.4°, θ_min_ = 0.8°
Absorption correction: multi-scan (SADABS; Krause *et al.*, 2015)	*h* = −4→4
*T*_min_ = 0.649, *T*_max_ = 0.746	*k* = −8→8
7403 measured reflections	*l* = −32→32
2824 independent reflections	

### (I) Refinement

**Table:**

Refinement on *F*^2^	Primary atom site location: dual
Least-squares matrix: full	Hydrogen site location: mixed
*R*[*F*^2^ > 2σ(*F*^2^)] = 0.086	H atoms treated by a mixture of independent and constrained refinement
*wR*(*F*^2^) = 0.212	*w* = 1/[σ^2^(*F*_o_^2^) + (0.0607*P*)^2^ + 3.3965*P*] where *P* = (*F*_o_^2^ + 2*F*_c_^2^)/3
*S* = 1.21	(Δ/σ)_max_ < 0.001
2824 reflections	Δρ_max_ = 0.96 e Å^−^^3^
241 parameters	Δρ_min_ = −0.53 e Å^−^^3^
1 restraint	

### (I) Special details

**Table:**

Geometry. All esds (except the esd in the dihedral angle between two l.s. planes) are estimated using the full covariance matrix. The cell esds are taken into account individually in the estimation of esds in distances, angles and torsion angles; correlations between esds in cell parameters are only used when they are defined by crystal symmetry. An approximate (isotropic) treatment of cell esds is used for estimating esds involving l.s. planes.

### (I) Fractional atomic coordinates and isotropic or equivalent isotropic displacement parameters (Å^2^)

**Table:**

	*x*	*y*	*z*	*U*_iso_*/*U*_eq_	
F1	−0.1924 (7)	0.3798 (3)	0.47161 (10)	0.0222 (6)	
O1	0.3060 (9)	0.3062 (4)	0.38931 (12)	0.0220 (7)	
N1	0.8293 (9)	0.6531 (5)	0.21239 (13)	0.0160 (7)	
C1	0.6976 (10)	0.4784 (6)	0.20518 (15)	0.0138 (7)	
F2	−0.3079 (8)	0.2526 (4)	0.57192 (10)	0.0242 (6)	
O2	0.0792 (9)	0.0469 (5)	0.35073 (11)	0.0221 (7)	
N2	0.5555 (9)	0.4571 (5)	0.30018 (13)	0.0161 (7)	
C2	0.5525 (10)	0.3755 (6)	0.25301 (15)	0.0143 (7)	
F3	1.1916 (6)	1.2914 (3)	0.06106 (9)	0.0153 (5)	
O3	1.1367 (8)	0.7643 (4)	0.12248 (11)	0.0184 (6)	
N3	0.7061 (10)	0.4043 (5)	0.15676 (14)	0.0180 (7)	
C3	0.6878 (11)	0.6381 (6)	0.30526 (16)	0.0170 (8)	
H3	0.684989	0.694550	0.339537	0.020*	
F4	1.3660 (6)	1.3695 (3)	−0.04072 (9)	0.0145 (5)	
O4	1.3969 (8)	1.0346 (4)	0.14728 (11)	0.0189 (6)	
N4	0.4156 (10)	0.1996 (5)	0.24889 (14)	0.0166 (7)	
C4	0.8238 (11)	0.7386 (6)	0.26166 (16)	0.0169 (8)	
H4	0.912708	0.864770	0.265250	0.020*	
C5	0.1481 (11)	0.1404 (6)	0.38960 (15)	0.0148 (7)	
C6	0.0608 (11)	0.0680 (6)	0.44630 (15)	0.0154 (8)	
C7	−0.0908 (11)	0.1937 (6)	0.48501 (15)	0.0167 (8)	
C8	−0.1498 (11)	0.1265 (6)	0.53729 (15)	0.0168 (8)	
C9	1.3066 (10)	0.9167 (5)	0.11367 (14)	0.0123 (7)	
C10	1.4083 (9)	0.9592 (5)	0.05484 (13)	0.0092 (7)	
C11	1.3511 (10)	1.1444 (5)	0.03174 (14)	0.0099 (7)	
C12	1.4395 (10)	1.1867 (5)	−0.02154 (14)	0.0099 (7)	
H4A	0.301 (16)	0.145 (8)	0.281 (2)	0.032 (15)*	
H1	0.919 (19)	0.711 (9)	0.1833 (18)	0.06 (2)*	
H4B	0.412 (16)	0.140 (8)	0.216 (2)	0.031 (14)*	
H3A	0.79 (2)	0.494 (10)	0.127 (3)	0.06 (2)*	
H1A	0.36 (2)	0.358 (11)	0.351 (3)	0.07 (2)*	
H3B	0.613 (15)	0.278 (8)	0.152 (2)	0.028 (14)*	

### (I) Atomic displacement parameters (Å^2^)

**Table:**

	*U*^11^	*U*^22^	*U*^33^	*U*^12^	*U*^13^	*U*^23^
F1	0.0313 (14)	0.0155 (12)	0.0203 (12)	−0.0040 (10)	−0.0026 (10)	−0.0021 (9)
O1	0.0314 (17)	0.0217 (15)	0.0140 (14)	−0.0128 (12)	−0.0002 (12)	0.0038 (11)
N1	0.0175 (16)	0.0169 (16)	0.0134 (15)	−0.0036 (12)	0.0005 (12)	0.0048 (12)
C1	0.0140 (18)	0.0150 (18)	0.0120 (17)	0.0024 (14)	0.0001 (13)	0.0011 (14)
F2	0.0352 (15)	0.0218 (13)	0.0158 (12)	−0.0024 (11)	0.0042 (10)	−0.0075 (10)
O2	0.0299 (17)	0.0268 (16)	0.0104 (13)	−0.0079 (13)	0.0010 (11)	−0.0028 (11)
N2	0.0172 (16)	0.0185 (16)	0.0122 (15)	−0.0004 (13)	0.0019 (12)	0.0021 (12)
C2	0.0119 (17)	0.0164 (18)	0.0142 (17)	−0.0011 (14)	−0.0005 (14)	0.0033 (14)
F3	0.0218 (12)	0.0115 (11)	0.0121 (10)	0.0038 (9)	0.0042 (9)	−0.0046 (8)
O3	0.0210 (14)	0.0193 (14)	0.0149 (13)	−0.0053 (11)	0.0029 (11)	0.0061 (11)
N3	0.0255 (18)	0.0158 (16)	0.0127 (15)	−0.0027 (13)	0.0042 (13)	0.0001 (12)
C3	0.0198 (19)	0.0178 (19)	0.0132 (18)	−0.0006 (15)	−0.0009 (15)	−0.0006 (14)
F4	0.0233 (12)	0.0063 (10)	0.0135 (10)	0.0012 (8)	0.0002 (9)	0.0034 (8)
O4	0.0254 (15)	0.0230 (15)	0.0088 (12)	−0.0024 (12)	−0.0010 (11)	−0.0031 (11)
N4	0.0235 (18)	0.0143 (16)	0.0123 (15)	−0.0043 (13)	0.0015 (13)	−0.0006 (12)
C4	0.0180 (19)	0.0149 (18)	0.0175 (19)	−0.0006 (14)	0.0005 (15)	0.0016 (14)
C5	0.0143 (18)	0.0190 (18)	0.0108 (17)	−0.0023 (14)	0.0015 (14)	0.0023 (14)
C6	0.0185 (19)	0.0205 (19)	0.0080 (16)	−0.0087 (15)	0.0003 (14)	−0.0008 (14)
C7	0.023 (2)	0.0159 (18)	0.0120 (17)	−0.0093 (15)	0.0001 (15)	−0.0010 (14)
C8	0.023 (2)	0.0171 (19)	0.0111 (17)	−0.0070 (15)	0.0007 (15)	−0.0046 (14)
C9	0.0130 (17)	0.0143 (17)	0.0093 (16)	0.0009 (13)	0.0010 (13)	0.0014 (13)
C10	0.0085 (15)	0.0137 (17)	0.0057 (15)	−0.0028 (13)	0.0005 (12)	−0.0003 (12)
C11	0.0109 (16)	0.0093 (16)	0.0097 (16)	−0.0010 (12)	0.0013 (13)	−0.0039 (12)
C12	0.0120 (16)	0.0072 (15)	0.0108 (16)	−0.0022 (12)	−0.0027 (13)	0.0026 (12)

### (I) Geometric parameters (Å, º)

**Table:**

F1—C7	1.339 (5)	N3—H3B	0.96 (6)
O1—C5	1.305 (5)	C3—C4	1.367 (5)
O1—H1A	1.02 (8)	C3—H3	0.9500
N1—C1	1.336 (5)	F4—C12	1.340 (4)
N1—C4	1.378 (5)	O4—C9	1.248 (5)
N1—H1	0.88 (2)	N4—H4A	0.96 (6)
C1—N3	1.325 (5)	N4—H4B	0.94 (6)
C1—C2	1.470 (5)	C4—H4	0.9500
F2—C8	1.336 (5)	C5—C6	1.516 (5)
O2—C5	1.220 (5)	C6—C7	1.396 (6)
N2—C2	1.320 (5)	C6—C8^i^	1.403 (6)
N2—C3	1.369 (5)	C7—C8	1.385 (5)
C2—N4	1.341 (5)	C9—C10	1.528 (5)
F3—C11	1.357 (4)	C10—C11	1.383 (5)
O3—C9	1.257 (5)	C10—C12^ii^	1.399 (5)
N3—H3A	1.01 (7)	C11—C12	1.386 (5)
			
C5—O1—H1A	112 (5)	O2—C5—C6	121.6 (3)
C1—N1—C4	122.7 (3)	O1—C5—C6	111.4 (3)
C1—N1—H1	115 (5)	C7—C6—C8^i^	117.2 (3)
C4—N1—H1	122 (5)	C7—C6—C5	121.7 (4)
N3—C1—N1	120.1 (3)	C8^i^—C6—C5	121.0 (4)
N3—C1—C2	123.0 (4)	F1—C7—C8	119.1 (4)
N1—C1—C2	116.9 (3)	F1—C7—C6	120.2 (3)
C2—N2—C3	121.0 (3)	C8—C7—C6	120.6 (4)
N2—C2—N4	119.9 (3)	F2—C8—C7	117.6 (4)
N2—C2—C1	119.8 (3)	F2—C8—C6^i^	120.2 (3)
N4—C2—C1	120.3 (3)	C7—C8—C6^i^	122.1 (4)
C1—N3—H3A	115 (4)	O4—C9—O3	127.3 (3)
C1—N3—H3B	120 (3)	O4—C9—C10	117.6 (3)
H3A—N3—H3B	124 (5)	O3—C9—C10	115.0 (3)
C4—C3—N2	120.9 (4)	C11—C10—C12^ii^	116.6 (3)
C4—C3—H3	119.6	C11—C10—C9	121.4 (3)
N2—C3—H3	119.6	C12^ii^—C10—C9	122.0 (3)
C2—N4—H4A	117 (3)	F3—C11—C10	119.9 (3)
C2—N4—H4B	121 (3)	F3—C11—C12	117.5 (3)
H4A—N4—H4B	122 (5)	C10—C11—C12	122.5 (3)
C3—C4—N1	118.7 (4)	F4—C12—C11	118.3 (3)
C3—C4—H4	120.7	F4—C12—C10^ii^	120.9 (3)
N1—C4—H4	120.7	C11—C12—C10^ii^	120.8 (3)
O2—C5—O1	126.9 (4)		
			
C4—N1—C1—N3	179.2 (4)	C5—C6—C7—C8	−176.5 (4)
C4—N1—C1—C2	−1.7 (6)	F1—C7—C8—F2	0.2 (6)
C3—N2—C2—N4	−178.8 (4)	C6—C7—C8—F2	−177.7 (3)
C3—N2—C2—C1	0.8 (6)	F1—C7—C8—C6^i^	178.0 (4)
N3—C1—C2—N2	179.4 (4)	C6—C7—C8—C6^i^	0.1 (7)
N1—C1—C2—N2	0.3 (5)	O4—C9—C10—C11	−45.5 (5)
N3—C1—C2—N4	−1.0 (6)	O3—C9—C10—C11	134.2 (4)
N1—C1—C2—N4	180.0 (4)	O4—C9—C10—C12^ii^	135.1 (4)
C2—N2—C3—C4	−0.6 (6)	O3—C9—C10—C12^ii^	−45.3 (5)
N2—C3—C4—N1	−0.7 (6)	C12^ii^—C10—C11—F3	177.5 (3)
C1—N1—C4—C3	2.0 (6)	C9—C10—C11—F3	−2.0 (5)
O2—C5—C6—C7	−137.7 (4)	C12^ii^—C10—C11—C12	−0.3 (6)
O1—C5—C6—C7	43.6 (5)	C9—C10—C11—C12	−179.7 (3)
O2—C5—C6—C8^i^	46.1 (6)	F3—C11—C12—F4	0.6 (5)
O1—C5—C6—C8^i^	−132.6 (4)	C10—C11—C12—F4	178.4 (3)
C8^i^—C6—C7—F1	−177.9 (3)	F3—C11—C12—C10^ii^	−177.5 (3)
C5—C6—C7—F1	5.7 (6)	C10—C11—C12—C10^ii^	0.3 (6)
C8^i^—C6—C7—C8	−0.1 (6)		

Symmetry codes: (i) −*x*, −*y*, −*z*+1; (ii) −*x*+3, −*y*+2, −*z*.

### (I) Hydrogen-bond geometry (Å, º)

**Table:**

*D*—H···*A*	*D*—H	H···*A*	*D*···*A*	*D*—H···*A*
C3—H3···F2^iii^	0.95	2.59	3.416 (5)	145
C4—H4···O2^iv^	0.95	2.61	3.307 (5)	130
N4—H4*A*···O2	0.96 (6)	2.02 (6)	2.980 (4)	175 (5)
N1—H1···O3	0.88 (2)	1.73 (3)	2.595 (4)	165 (7)
N4—H4*B*···O4^v^	0.94 (6)	1.88 (6)	2.814 (4)	177 (5)
N3—H3*A*···F3^vi^	1.01 (7)	2.54 (7)	3.027 (4)	109 (5)
N3—H3*A*···O3	1.01 (7)	2.30 (7)	3.091 (4)	134 (5)
N3—H3*A*···F4^vii^	1.01 (7)	2.39 (7)	3.249 (4)	143 (6)
O1—H1*A*···N2	1.02 (8)	1.60 (8)	2.589 (4)	162 (7)
N3—H3*B*···O4^v^	0.96 (6)	1.89 (6)	2.855 (5)	174 (5)

Symmetry codes: (iii) −*x*, −*y*+1, −*z*+1; (iv) *x*+1, *y*+1, *z*; (v) *x*−1, *y*−1, *z*; (vi) *x*, *y*−1, *z*; (vii) −*x*+2, −*y*+2, −*z*.
